# Fetal Macrosomia and Postpartum Hemorrhage in Latin American and Caribbean Region: Systematic Review and Meta-analysis

**DOI:** 10.1055/s-0043-1772597

**Published:** 2023-11-29

**Authors:** Araceli Quezada-Robles, Fiorella Quispe-Sarmiento, Guido Bendezu-Quispe, Rodrigo Vargas-Fernández

**Affiliations:** 1 Universidad Científica del Sur, Facultad de Ciencias de la Salud, Lima, Peru; 2 Universidad César Vallejo. Escuela de Medicina. Trujillo, Perú

**Keywords:** postpartum hemorrhage, fetal macrosomia, systematic review, meta-analysis, Latin America, hemorragia pós-parto, macrossomia fetal, revisão sistemática, metanálise, América latina

## Abstract

**Objective** To determine the association between fetal macrosomia (FM) and postpartum hemorrhage (PPH) in Latin American and Caribbean (LAC) women.

**Data Sources** Studies evaluating the association between FM and PPH (≥ 500 ml) and severe PPH (≥ 1,000 ml) until November 4, 2021, indexed in CINHAL, Scopus, Embase, Cochrane Library, MEDLINE, LILACS, and SciELO.

**Selection of Studies** Inclusion criteria were cohort and case-control studies that provided the number of PPH and FM cases. Exclusion criteria were studies lacking information about the number of cases, with a population of women who were not from LAC; published in a language other than English, Spanish, or Portuguese, and with a different design.

**Data Collection** Data extraction was performed independently by two authors, and discrepancies were resolved with a third author. Data regarding FM and PPH cases were retrieved.

**Data Synthesis** Of the 1,044 articles evaluated, 5 studies were included, from 6 different countries: Argentina and Uruguay (multi-country), West Indies, Antigua and Barbuda, French Guyana, and Suriname. The pooled odds ratio (OR) for FM and PPH in the meta-analysis (five studies) was 2.10 (95% confidence interval [CI]: 1.79–2.47; I ^2^ : 0%), with estimates within this 95% CI in the sensitivity analysis. The combined OR for severe PPH (3 studies) was 1.61 (95% CI: 0.40–6.48; I ^2^ : 91.89%), showing high heterogeneity.

**Conclusion** There was a positive association between FM and PPH in the LAC, increasing the risk of the presence of this event 2-fold. The high heterogeneity of the studies that measured severe PPH does not allow drawing conclusions about the estimates obtained.

## Resumo

**Objetivo** Determinar a associação entre macrossomia fetal (FM) e hemorragia pós-parto (HPP) em mulheres da América Latina e Caribe (ALC).

**Fontes de dados** Estudos avaliando a associação entre FM e HPP (≥ 500 ml) e HPP grave (≥ 1.000 ml) até 4 de novembro de 2021, indexados no CINHAL, Scopus, Embase, Biblioteca Cochrane, MEDLINE, LILACS e SciELO.

**Seleção de estudos** Os critérios de inclusão foram estudos de corte e caso-controle que forneceram o número de casos de HPP e FM. Os critérios de exclusão foram estudos sem informação sobre o número de casos, com uma população de mulheres que não eram da ALC; publicado em um idioma diferente do inglês, espanhol ou português e com um design diferente.

**Coleta de dados** A extração de dados foi realizada independentemente por dois autores, as discrepâncias foram resolvidas com um terceiro autor. Os dados relativos aos casos de FM e HPP foram recuperados.

**Síntese dos dados** Dos 1.044 artigos avaliados, foram incluídos 5 estudos, de 6 países diferentes: Argentina e Uruguai (multipaíses), Índias Ocidentais, Antígua e Barbuda, Guiana Francesa e Suriname. O *odds ratio* agrupado (OR) para FM e HPP na meta-análise (cinco estudos) foi de 2,10 (intervalo de confiança de 95% [IC]: 1,79–2,47; I ^2^ : 0%), com estimativas dentro deste IC de 95% no análise sensitiva. O OR combinado para HPP grave (3 estudos) foi de 1,61 (95% CI: 0.40–6.48; I ^2^ : 91.89%), mostrando alta heterogeneidade.

**Conclusão** Houve associação positiva entre FM e HPP na ALC, aumentando em 2 vezes o risco da presença desse evento. A alta heterogeneidade dos estudos que mediram a HPP grave não permite tirar conclusões sobre as estimativas obtidas.

## Introduction

Postpartum hemorrhage (PPH) is a public health problem. It is traditionally defined as blood volume loss greater than 500 ml after vaginal delivery or 1,000 ml after cesarean delivery. Recently, the American College of Obstetricians and Gynecologists has defined PPH as a cumulative blood volume loss ≥ 1,000 ml or blood loss associated with clinical manifestations of hypovolemia (such as hypotension and tachycardia), regardless of the route of delivery. 1 Globally, PPH is the leading cause of maternal mortality, with over 80,000 deaths in 2015, with low and middle-income countries presenting more than 30 times the number of maternal deaths compared with high-income ones. 2 Additionally, the global prevalence of PPH (≥ 500 ml) exceeds 10% of women giving birth, with the highest rates being found in Africa (25.7%), North America (13.1%), and Europe (12.7%). Furthermore, Africa (5.1%), North America (4.3%), and Latin America and the Caribbean (LAC, 3.3%) present the highest prevalence rates of severe PPH (≥ 1,000 ml). 3

Globally, the main causes of PPH are uterine atony (more than 70%), obstetric lacerations (20%), retained placental tissue (10%) and clotting factor deficiencies (less than 1%). 1 In turn, fetal macrosomia (FM, gestational weight ≥ 4,000 g) is recognized as a risk factor for the occurrence of PPH in several regions of the world, 4 5 6 being mainly associated with preexisting diabetes, maternal obesity before pregnancy, gestational diabetes, excessive weight gain during gestation, abnormal fasting and postprandial glucose levels, dyslipidemia, history of a macrosomic fetus, and postterm pregnancy. 7 Proper identification of the predictors of PPH, as well as active management of stage 3 of labor, is crucial for the prevention of this health problem, which is still the leading cause of maternal death in low- and middle-income countries.

The countries that make up the LAC region have high fertility rates, high levels of poverty, and poor health care coverage and quality, which have resulted in a maternal mortality rate in this region of 88 maternal deaths per 100,000 live births. 8 The prevalence of FM in LAC varies between 4.5 and 5.4%. 9 An increase in obesity and diabetes in women has been described in this region, 10 which could explain the increase in the prevalence of FM. 9 Despite the knowledge about PPH and FM in various regions of the world, there is still little evidence about its association in LAC. Therefore, the aim of the present study was to determine the association between FM and PPH in women from LAC through a systematic review with meta-analysis of the published scientific literature.

## Methods

The systematic review protocol was registered in the Prospective International Registry of Systematic Reviews (PROSPERO) (CRD42021233589). This study followed the Preferred Reporting Items for Systematic Reviews and Metanalyses (PRISMA) 2020 guidelines. 11 This systematic review with meta-analysis focused on studies conducted in populations from LAC.

On November 14, 2021, a comprehensive search for studies that made estimates of the association between FM and PPH in women from LAC was conducted using seven electronic bibliographic databases: CINHAL, Scopus, Embase, Cochrane Library, MEDLINE, LILACS, and SciELO. The search terms focused on postpartum hemorrhage, fetal macrosomia, Latin America, and Caribbean Region. The countries considered as part of LAC in this study are those included on the Pan-American Health Organization (PAHO) list. 12 The electronic search did not require additional language, time, or design filters and was complemented by a manual review of the references of the included articles ( Chart 1 ). The records found in the electronic search were imported to the Mendeley (Elsevier, Amsterdam, Netherlands) reference management software, and all duplicate records were removed.

**Chart 1:** Search Strategies

**Database**	**MEDLINE** **Date: November 4, 2021**		**Results**
**Search Strategy**	#1	Postpartum Hemorrhage[Mesh] OR Shock, Hemorrhagic[Mesh] OR Uterine Inertia[Mesh] OR Uterine Hemorrhage[Mesh] OR Abruptio Placentae[Mesh] OR Blood Loss, Surgical[Mesh] OR Blood Transfusion[Mesh] OR Placenta Accreta[Mesh] OR Placenta Previa[Mesh] OR uterine inversion[Mesh] OR Uterine Artery Embolization[Mesh] OR Uterine Contraction[Mesh] OR Obstetric Labor Complications[Mesh] OR Postpartum Hemorrhag*[tiab] OR Postpartum Haemorrhag*[tiab] OR post partum hemorrhage[tiab] OR post-partum hemorrhage[tiab] OR PPH[tiab] OR Abruptio Placentae[tiab] OR abruption[tiab] OR blood loss*[tiab] OR blood transfusion[tiab] OR placenta accreta[tiab] OR placenta previa[tiab] OR shock[tiab] OR placenta praevia[tiab] OR placental previa[tiab] OR placental praevia[tiab] OR atony[tiab] OR atonic[tiab] OR atonic uterus[tiab] OR uterine atony[tiab] OR uterine inertia[tiab] OR uterine bleeding[tiab] OR uterine hemorrhage[tiab] OR uterine hemorrhage[tiab] OR uterus inversion[tiab] OR uterine artery embolization[tiab] OR uterine contraction[tiab] OR labor complication*[tiab] OR labor complication*[tiab] OR delivery complication*[tiab]	467,480
	#2	Fetal Macrosomia[Mesh] OR Fetal Disease[Mesh] OR Fetal Macrosomia[tiab] OR Macrosom*[tiab] OR fetal overgrowth[tiab] OR large-for-gestational-age[tiab] OR Fetal complication*[tiab]	77,119
	#3	#1 AND #2	11,950
	#4	Americas[MeSH Terms:noexp] OR America*[tiab] OR Latin America[Mesh] OR Latin America*[tiab] OR Latinamerica*[tiab] OR Latinoamerica*[tiab] OR Latin*[tiab] OR Hispanic Americans[Mesh] OR Hispanic America*[tiab] OR Hispanoamerica*[tiab] OR Iberoamerica*[tiab] OR Ibero Americ*[tiab] OR Panamerican*[tiab] OR Central America[Mesh] OR Central America*[tiab] OR Centroamerica*[tiab] OR Mesoamerica*[tiab] OR Meso America*[tiab] OR Middle America*[tiab] OR South America[Mesh] OR South America*[tiab] OR Southamerica*[tiab] OR Sudamerica*[tiab] OR “America del sur”[tiab] OR Caribbean Region[Mesh] OR Caribbean[tiab] OR Caribe*[tiab] OR West Indies[Mesh] OR West Indi*[tiab] OR Antill*[tiab] OR Indians, South American[Mesh] OR Indians, Central American[Mesh] OR Amerindian*[tiab] OR Indians[tiab] OR American Indian*[tiab] OR Native America*[tiab] OR Patagoni*[tiab] OR Andes[tiab] OR Andean*[tiab] OR Amazon*[tiab] OR Argentin*[ad] OR Argentin*[tiab] OR Argentina[pl] OR Bolivia*[ad] OR Bolivia*[tiab] OR Bolivia[pl] OR Brazil*[ad] OR Brasil*[ad] OR Brazil*[tiab] OR Brasil*[tiab] OR Brazil[pl] OR Colombia*[ad] OR Colombia*[tiab] OR Colombia[pl] OR Chile*[ad] OR Chile*[tiab] OR Chile[pl] OR Ecuador*[ad] OR Ecuator*[ad] OR Ecuador*[tiab] OR Ecuador[pl] OR Guiana*[ad] OR Guiana*[tiab] OR French Guiana[pl] OR Guyan*[ad] OR Guyan*[tiab] OR Guyana[pl] OR Paraguay*[ad] OR Paraguay*[tiab] OR Paraguay[pl] OR Peru*[ad] OR Peru*[tiab] OR Peru[pl] OR Surinam*[ad] OR Surinam*[tiab] OR Suriname[pl] OR Uruguay*[ad] OR Uruguay*[tiab] OR Uruguay[pl] OR Venez*[ad] OR Venez*[tiab] OR Venezuela[pl] OR Belize*[ad] OR Belize*[tiab] OR Belize[pl] OR Costa Ric*[ad] OR Costarric*[ad] OR Costaric*[ad] OR Costa Ric*[tiab] OR Costarric*[tiab] OR Costaric*[tiab] OR Costa Rica[pl] OR Salvador*[ad] OR Salvador*[tiab] OR El Salvador[pl] OR Guatemal*[ad] OR Guatemal*[tiab] OR Guatemala[pl] OR Hondur*[ad] OR Hondur*[tiab] OR Honduras[pl] OR Nicaragu*[ad] OR Nicaragu*[tiab] OR Nicaragua[pl] OR Panam*[ad] OR Panam*[tiab] OR Panama[pl] OR Mexico[Mesh] OR Mexic*[ad] OR Mexic*[tiab] OR Mejic*[tiab] OR Mexico[pl] OR Aruba*[ad] OR Aruba*[tiab] OR Aruba[pl] OR Caribbean Netherland*[ad] OR Caribbean Netherland*[tiab] OR Caribbean Netherlands[pl] OR Curacao*[ad] OR Curacao*[tiab] OR Curacao[pl] OR “Sint Maarten”[ad] OR “Sint Maarten”[tiab] OR “Sint Maarten”[pl] OR Guadeloup*[ad] OR Guadeloup*[tiab] OR Guadeloupe[pl] OR Martiniqu*[ad] OR Martiniqu*[tiab] OR Martinique[pl] OR “Panama Canal Zone”[ad] OR “Panama Canal Zone”[tiab] OR “Panama Canal Zone”[pl] OR “Antigua and Barbuda”[ad] OR “Antigua and Barbuda”[tiab] OR “Antigua and Barbuda”[pl] OR Baham*[ad] OR Baham*[tiab] OR Bahamas[pl] OR Barbad*[ad] OR Barbad*[tiab] OR Barbados[pl] OR “British Virgin Island”[ad] OR “British Virgin Island”[tiab] OR “British Virgin Islands”[pl] OR Cayman Island*[ad] OR Cayman Island*[tiab] OR Cayman Islands[pl] OR Grenad*[ad] OR Grenad*[tiab] OR Grenada[pl] OR “Saint Kitts and Nevis”[ad] OR “Saint Kitts and Nevis”[tiab] OR “Saint Kitts and Nevis”[pl] OR Saint Lucia*[ad] OR Saint Lucia*[tiab] OR Saint Lucia[pl] OR “Saint Vincent and the Grenadines”[ad] OR “Saint Vincent and the Grenadines”[tiab] OR Saint Vincent and the Grenadines[pl] OR “Trinidad and Tobago”[ad] OR “Trinidad and Tobago”[tiab] OR “Trinidad and Tobago”[pl] OR “Turks and Caicos Island”[ad] OR “Turks and Caicos Island”[tiab] OR “Turks and Caicos Islands”[pl] OR “United States Virgin Islands”[ad] OR “United States Virgin Islands”[tiab] OR “United States Virgin Islands”[pl] OR Anguill*[ad] OR Anguill*[tiab] OR Anguilla[pl] OR Leeward Island*[ad] OR Leeward Island*[tiab] OR Leeward Islands[pl] OR Montserrat*[ad] OR Montserrat*[tiab] OR Montserrat[pl] OR Windward Island*[ad] OR Windward Island*[tiab] OR Windward Islands[pl] OR Cuba*[ad] OR Cuba*[tiab] OR Cuba[pl] OR Dominic*[ad] OR Dominic*[tiab] OR Dominican Republic[pl] OR Haiti*[ad] OR Haiti*[tiab] OR Haiti[pl] OR Jamaic*[ad] OR Jamaic*[tiab] OR Jamaica[pl] OR Puerto Ric*[tiab] OR Puertorric*[tiab] OR Puertoric*[tiab]	1,662,291
	#5	#3 AND #4	556
**Database**	**Embase** **Date: November 4, 2021**		**Results**
**Search Strategy**	#1	'postpartum hemorrhage'/exp OR 'postpartum hemorrhage' OR 'hemorrhagic shock'/exp OR 'hemorrhagic shock' OR 'uterus bleeding'/exp OR 'uterus bleeding' OR 'solutio placentae'/exp OR 'solutio placentae' OR 'bleeding'/exp OR 'bleeding' OR 'operative blood loss'/exp OR 'operative blood loss' OR 'blood transfusion'/exp OR 'blood transfusion' OR 'placenta accreta'/exp OR 'placenta accreta' OR 'placenta previa'/exp OR 'placenta previa' OR 'uterus inversion'/exp OR 'uterus inversion' OR 'uterine artery embolization'/exp OR 'uterine artery embolization' OR 'uterus contraction'/exp OR 'uterus contraction' OR 'abruptio placentae':ti,ab OR 'abruption':ti,ab OR 'surgical blood loss':ti,ab OR 'blood loss':ti,ab OR 'blood transfusion':ti,ab OR 'placenta accreta':ti,ab OR 'shock':ti,ab OR 'haemorrhagic shock':ti,ab OR 'hemorrhagic shock':ti,ab OR 'placental accreta':ti,ab OR 'placenta previa':ti,ab OR 'placenta praevia':ti,ab OR 'placental previa':ti,ab OR 'placental praevia':ti,ab OR 'pph':ti,ab OR 'postpartum hemorrhage':ti,ab OR 'postpartum hemorrhage':ti,ab OR 'post partum hemorrhage':ti,ab OR 'post partum hemorrhage':ti,ab OR 'post-partum hemorrhage':ti,ab OR 'post-partum hemorrhage':ti,ab OR 'atony':ti,ab OR 'atonic':ti,ab OR 'atonic uterus':ti,ab OR 'uterine atony':ti,ab OR 'uterine inertia':ti,ab OR 'uterine bleeding':ti,ab OR 'uterine hemorrhage':ti,ab OR 'uterine hemorrhage':ti,ab OR 'labor complication':ti,ab OR 'delivery complication':ti,ab OR 'labor complication':ti,ab OR 'uterus inversion':ti,ab OR 'uterine artery embolization':ti,ab OR 'uterine contraction':ti,ab	1,590,724
	#2	'macrosomia'/exp OR 'macrosom*:ti,ab' OR 'fetus disease'/exp OR 'fetal macrosomia:ti,ab' OR 'fetal overgrowth:ti,ab' OR 'large for gestational age:ti,ab' OR 'fetal complication:ti,ab'	132,774
	#3	#1 AND #2	18,763
	#4	americas:ti,ab OR 'south and central america'/exp OR ((latin NEAR/1 america*):ti,ab) OR latinamerica*:ti,ab OR latinoamerica*:ti,ab OR hispanoamerica:ti,ab OR iberoamerica*:ti,ab OR ((ibero NEAR/1 americ*):ti,ab) OR panamerica*:ti,ab OR ((south NEAR/1 america*):ti,ab) OR southamerica*:ti,ab OR sudamerica*:ti,ab OR (america:ti,ab AND del:ti,ab AND sur:ti,ab) OR ((central NEAR/1 america*):ti,ab) OR centroamerica*:ti,ab OR mesoamerica*:ti,ab OR ((meso NEAR/1 america*):ti,ab) OR ((middle NEAR/1 america*):ti,ab) OR 'caribbean'/exp OR 'caribbean islands'/exp OR caribbean*:ti,ab OR caribe*:ti,ab OR ((west NEAR/1 indi*):ti,ab) OR antill*:ti,ab OR 'american indian'/exp OR amerindian*ti,ab OR indians:ti,ab OR ((native NEAR/1 america*):ti,ab) OR patagoni*:ti,ab OR andes:ti,ab OR andean*:ti,ab OR amazon*:ti,ab OR 'argentina'/exp OR argentin*:ti,ab OR 'bolivia'/exp OR bolivia*:ti,ab OR 'brazil'/exp OR brazil*:ti,ab OR brasil*:ti,ab OR 'colombia'/exp OR colombia*:ti,ab OR 'chile'/exp OR chile*:ti,ab OR 'ecuador'/exp OR ecuador*:ti,ab OR 'french guiana'/exp OR guiana*:ti,ab OR 'guyana'/exp OR guyan*:ti,ab OR 'paraguay'/exp OR paraguay*:ti,ab OR 'peru'/exp OR peru*:ti,ab OR 'suriname'/exp OR surinam*:ti,ab OR 'uruguay'/exp OR uruguay*:ti,ab OR 'venezuela'/exp OR venez*:ti,ab OR 'belize'/exp OR beliz*:ti,ab OR 'costa rica'/exp OR 'costa rica':ti,ab OR costarric*:ti,ab OR costaric*:ti,ab OR 'el salvador'/exp OR salvador*:ti,ab OR 'guatemala'/exp OR guatemal*:ti,ab OR 'honduras'/exp OR hondur*:ti,ab OR 'nicaragua'/exp OR nicaragu*:ti,ab OR 'panama'/exp OR panam*:ti,ab OR 'mexico'/exp OR mexic*:ti,ab OR mejic*:ti,ab OR 'cuba'/exp OR cuba*:ti,ab OR 'dominican republic'/exp OR dominica*:ti,ab OR 'haiti'/exp OR haiti*:ti,ab OR 'jamaica'/exp OR jamaic*:ti,ab OR 'puerto rico'/exp OR ((puerto NEAR/1 ric*):ti,ab) OR puertoric*:ti,ab OR puertorric*:ti,ab	575,956
	#5	#3 AND #4	264
**Database**	**CINAHL Complete** **Date: November 4, 2021**		**Results**
**Search Strategy**	S1	(MH “Postpartum Hemorrhage”)	3,869
	S2	(MH “Shock, Hemorrhagic”)	2,094
	S3	(MH “Uterine Inertia”)	253
	S4	(MH “Uterine Hemorrhage”)	2,151
	S5	(MH “Abruptio Placentae”)	809
	S6	(MH “Blood Loss, Surgical”)	5,892
	S7	(MH “Blood Transfusion”)	13,703
	S8	(MH “Placenta Accreta”)	1,190
	S9	(MH “Placenta Previa”)	1,091
	S10	(MH “uterine inversion”)	96
	S11	(MH “Uterine Artery Embolization”)	666
	S12	(MH “Uterine Contraction”)	1,055
	S13	(MH “Obstetric Labor Complications”)	2,338
	S14	TI (Postpartum N1 Hemorrhag*) OR AB (Postpartum N1 Hemorrhag*)	2,221
	S15	TI (Postpartum N1 Haemorrhag*) OR AB (Postpartum N1 Haemorrhag*)	941
	S16	TI (post-partum hemorrhage*) OR AB (post-partum hemorrhage*)	222
	S17	TI (post-partum N1 hemorrhage*) OR AB (post-partum N1 hemorrhage*)	192
	S18	TI PPH OR AB PPH	1,155
	S19	TI (Abruptio N1 Placentae) OR AB (Abruptio N1 Placentae)	202
	S20	TI abruption OR AB abruption	1,165
	S21	TI (blood N1 loss*) OR AB (blood N1 loss*)	13,587
	S22	TI (blood N1 transfusion) OR AB (blood N1 transfusion)	12,005
	S23	TI (placenta N1 accreta) OR AB (placenta N1 accreta)	1,089
	S24	TI (placenta N1 previa) OR AB (placenta N1 previa)	1,042
	S25	TI shock OR AB shock	30,290
	S26	TI (placenta N1 praevia) OR AB (placenta N1 praevia)	200
	S27	TI (placental N1 previa) OR AB (placental N1 previa)	60
	S28	TI (placental N1 praevia) OR AB (placental N1 praevia)	20
	S29	TI atony OR AB atony	349
	S30	TI atonic OR AB atonic	212
	S31	TI (atonic N1 uterus) OR AB (atonic N1 uterus)	17
	S32	TI (uterine N1 atony) OR AB (uterine N1 atony)	293
	S33	TI (uterine N1 inertia) OR AB (uterine N1 inertia)	15
	S34	TI (uterine N1 bleeding) OR AB (uterine N1 bleeding)	1,291
	S35	TI (uterine N1 hemorrhage) OR AB (uterine N1 hemorrhage)	136
	S36	TI (uterine N1 hemorrhage) OR AB (uterine N1 hemorrhage)	136
	S37	TI (uterus N1 inversion) OR AB (uterus N1 inversion)	4
	S38	TI “uterine artery embolization” OR AB “uterine artery embolization”	861
	S39	TI (uterine N1 contraction) OR AB (uterine N1 contraction)	778
	S40	TI labor complication* OR AB labor complication*	725
	S41	TI labor complication* OR AB labor complication*	725
	S42	TI delivery complication* OR AB delivery complication*	1,899
	S43	S1 OR S2 OR S3 OR S4 OR S5 OR S6 OR S7 OR S8 OR S9 OR S10 OR S11 OR S12 OR S13 OR S14 OR S15 OR S16 OR S17 OR S18 OR S19 OR S20 OR S21 OR S22 OR S23 OR S24 OR S25 OR S26 OR S27 OR S28 OR S29 OR S30 OR S31 OR S32 OR S33 OR S34 OR S35 OR S36 OR S37 OR S38 OR S39 OR S40 OR S41 OR S42	77,892
	S44	(MH “Fetal Macrosomia”)	1,288
	S45	(MH “Fetal Disease”)	2,991
	S46	TI (Fetal N1 Macrosomia) OR AB (Fetal N1 Macrosomia)	358
	S47	TI Macrosom* OR AB Macrosom*	1,648
	S48	TI (fetal N1 overgrowth) OR AB (fetal N1 overgrowth)	91
	S49	TI large-for-gestational-age OR AB large-for-gestational-age	1,297
	S50	TI Fetal complication* OR AB Fetal complication*	1,349
	S51	S44 OR S45 OR S46 OR S47 OR S48 OR S49 OR S50	4,369
	S52	S43 AND 51	517
	S53	TI (Latin America* OR Latinamerica* OR Latinoamerica* OR Latin* OR Hispanic Americans OR Iberoamerica* OR Ibero Americ* OR Panamerican* OR Central America* OR Centroamerica* OR Mesoamerica* OR Meso America* OR Middle America* OR South America* OR Southamerica* OR Sudamerica* OR America del sur OR Caribbean OR Caribe* OR West Indi* OR Antill* OR Amerindian* OR Indians OR American Indian* OR Native America* OR Patagoni* OR Andes OR Andean* OR Amazon* OR Argentin* OR Bolivia* OR Brazil* OR Brasil* Colombia* OR Colombia* OR Colombia OR Chile* OR Ecuador* OR Guiana* OR Guyan* OR Guyan* OR Paraguay* OR Paraguay* OR Peru* OR Surinam* OR Surinam* OR Uruguay* OR Venez* OR Belize* OR Costa Ric* OR Costarric* OR Costaric* OR Costa Ric* OR Costarric* OR Salvador* OR Salvador* OR El Salvador OR Guatemal* OR Guatemal* OR Guatemala OR Hondur* OR Nicaragu* Panam* OR Mexic* OR Cuba* OR Dominic* OR Dominic* OR Haiti* OR Jamaic* OR Puerto Ric* OR Puertorric* OR Puertoric*)	66,056
	S54	AB (Latin America* OR Latinamerica* OR Latinoamerica* OR Latin* OR Hispanic Americans OR Iberoamerica* OR Ibero Americ* OR Panamerican* OR Central America* OR Centroamerica* OR Mesoamerica* OR Meso America* OR Middle America* OR South America* OR Southamerica* OR Sudamerica* OR America del sur OR Caribbean OR Caribe* OR West Indi* OR Antill* OR Amerindian* OR Indians OR American Indian* OR Native America* OR Patagoni* OR Andes OR Andean* OR Amazon* OR Argentin* OR Bolivia* OR Brazil* OR Brasil* Colombia* OR Colombia* OR Colombia OR Chile* OR Ecuador* OR Guiana* OR Guyan* OR Guyan* OR Paraguay* OR Paraguay* OR Peru* OR Surinam* OR Surinam* OR Uruguay* OR Venez* OR Belize* OR Costa Ric* OR Costarric* OR Costaric* OR Costa Ric* OR Costarric* OR Salvador* OR Salvador* OR El Salvador OR Guatemal* OR Guatemal* OR Guatemala OR Hondur* OR Nicaragu* Panam* OR Mexic* OR Cuba* OR Dominic* OR Dominic* OR Haiti* OR Jamaic* OR Puerto Ric* OR Puertorric* OR Puertoric*)	99,564
	S55	S53 OR S54	125,766
	S56	S52 AND S54	10
**Database**	**Scopus** **Date: November 4, 2021**		**Results**
**Search Strategy**	#1	TITLE-ABS-KEY (“Postpartum Hemorrhage”)) OR (TITLE-ABS-KEY (“Shock, Hemorrhagic”)) OR (TITLE-ABS-KEY (“Uterine Inertia”)) OR (TITLE-ABS-KEY (“Uterine Hemorrhage”)) OR (TITLE-ABS-KEY (“Uterine Hemorrhage”)) OR (TITLE-ABS-KEY (“Abruptio Placentae”)) OR (TITLE-ABS-KEY (“Blood Loss, Surgical”)) OR (TITLE-ABS-KEY (“Blood Transfusion”)) OR (TITLE-ABS-KEY (“Placenta Accreta”)) OR (TITLE-ABS-KEY (“Placenta Previa”)) OR (TITLE-ABS-KEY (“uterine inversion”)) OR (TITLE-ABS-KEY (“Uterine Artery Embolization”)) OR (TITLE-ABS-KEY (“Uterine Contraction”)) OR (TITLE-ABS-KEY (“Obstetric Labor Complication*”)) OR (TITLE-ABS-KEY (“Postpartum Haemorrhag*”)) OR (TITLE-ABS-KEY (“post-partum haemorrhage”)) OR (TITLE-ABS-KEY (“post-partum hemorrhage”)) OR (TITLE-ABS-KEY (pph)) OR (TITLE-ABS-KEY (abruption)) OR (TITLE-ABS-KEY (shock)) OR (TITLE-ABS-KEY (“placenta praevia”)) OR (TITLE-ABS-KEY (“placental previa”)) OR (TITLE-ABS-KEY (“placental praevia”)) OR (TITLE-ABS-KEY (atony)) OR (TITLE-ABS-KEY (atonic)) OR (TITLE-ABS-KEY (“atonic uterus”)) OR (TITLE-ABS-KEY (“uterine atony”)) OR (TITLE-ABS-KEY (“uterine inertia”)) OR (TITLE-ABS-KEY (“uterine bleeding”)) OR (TITLE-ABS-KEY (“uterine haemorrhage”))) OR (TITLE-ABS-KEY (“uterus inversion”)) OR (TITLE-ABS-KEY (“labor complication*”)) OR (TITLE-ABS-KEY (“labour complication*”)) OR (TITLE-ABS-KEY (“delivery complication*”)	839,680
	#2	TITLE-ABS-KEY (“Fetal Macrosomia”)) OR (TITLE-ABS-KEY (“Fetal Disease*”)) OR (TITLE-ABS-KEY (macrosom*)) OR (TITLE-ABS-KEY (“fetal overgrowth”)) OR (TITLE-ABS-KEY (“large-for-gestational-age”)) OR (TITLE-ABS-KEY (“Fetal complication*”)	38,064
	#3	#1 AND #2	
	#4	TITLE-ABS-KEY (“latin america” OR “Latinoamerica” OR latin* OR “central america” OR “Centroamerica” OR “south America” OR sudamerica OR caribbean OR caribe* OR “west indies” OR antill* OR patagoni* OR andes OR andean OR amazon OR “Puerto rico” OR puertoric* OR puertorric* OR jamaica OR jamaic* OR haiti OR haiti* OR “dominican republic” OR dominica* OR cuba OR cuba* OR mexico OR mexic* OR mejic* OR panama OR panam* OR nicaragua OR nicaragu* OR honduras OR hondur* OR guatemala OR guatemal* OR “el Salvador” OR salvador* OR “costa rica” OR costarric* OR costaric* OR belize OR beliz* OR venezuela OR venez* OR uruguay OR uruguay* OR suriname OR surinam* OR peru OR peru* OR paraguay OR paraguay* OR guyana OR guyan* OR “french guiana” OR guiana* OR guayan* OR ecuador OR ecuador* OR chile OR chile* OR colombia OR colombia* OR brazil OR brazil* OR brasil* OR bolivia OR bolivia* OR argentina OR argentin*	1,414,676
	#5	#3 AND #4	38
**Database**	**The Cochrane Library** **Date: November 4, 2021**		**Results**
**Search Strategy**	#1	MeSH descriptor: [Postpartum Hemorrhage] explode all trees	697
	#2	MeSH descriptor: [Shock, Hemorrhagic] explode all trees	111
	#3	MeSH descriptor: [Uterine Inertia] explode all trees	47
	#4	MeSH descriptor: [Uterine Hemorrhage] explode all trees	1,871
	#5	MeSH descriptor: [Abruptio Placentae] explode all trees	31
	#6	MeSH descriptor: [Blood Loss, Surgical] explode all trees	2,749
	#7	MeSH descriptor: [Blood Transfusion] explode all trees	3,681
	#8	MeSH descriptor: [Placenta Accreta] explode all trees	31
	#9	MeSH descriptor: [Placenta Previa] explode all trees	63
	#10	MeSH descriptor: [Uterine Inversion] explode all trees	0
	#11	MeSH descriptor: [Uterine Artery Embolization] explode all trees	55
	#12	MeSH descriptor: [Uterine Contraction] explode all trees	381
	#13	MeSH descriptor: [Obstetric Labor Complications] explode all trees	4,162
	#14	(Postpartum Hemorrhag*):ti,ab,kw	2,002
	#15	(Postpartum Haemorrhag*):ti,ab,kw	761
	#16	(post-partum hemorrhage):ti,ab,kw	415
	#17	(post-partum hemorrhage):ti,ab,kw	413
	#18	(PPH):ti,ab,kw	768
	#19	(Abruptio Placentae):ti,ab,kw	81
	#20	(abruption):ti,ab,kw	424
	#21	(blood loss*):ti,ab,kw	33,271
	#22	(blood transfusion):ti,ab,kw	13,489
	#23	(placenta accreta):ti,ab,kw	112
	#24	(placenta previa):ti,ab,kw	379
	#25	(shock):ti,ab,kw	11,523
	#26	(placenta praevia):ti,ab,kw	52
	#27	(placental previa):ti,ab,kw	110
	#28	(placental praevia):ti,ab,kw	13
	#29	(atony):ti,ab,kw	259
	#30	(atonic):ti,ab,kw	171
	#31	(atonic uterus):ti,ab,kw	32
	#32	(uterine atony):ti,ab,kw	217
	#33	(uterine inertia):ti,ab,kw	60
	#34	(uterine bleeding):ti,ab,kw	3,029
	#35	(uterine hemorrhage):ti,ab,kw	1,801
	#36	(uterine hemorrhage):ti,ab,kw	1,801
	#37	(uterus inversion):ti,ab,kw	14
	#38	(uterine artery embolization):ti,ab,kw	294
	#39	(uterine contraction):ti,ab,kw	1,053
	#40	(labor complication*):ti,ab,kw	3,563
	#41	(labor complication*):ti,ab,kw	3,560
	#42	(delivery complication*):ti,ab,kw	8,252
	#43	#1 OR #2 OR #3 OR #4 OR #5 OR #6 OR #7 OR #8 OR #9 OR #10 OR #11 OR #12 OR #13 OR #14 OR #15 OR #16 OR #17 OR #18 OR #19 OR #20 OR #21 OR #22 OR #23 OR #24 OR #25 OR #26 OR #27 OR #28 OR #29 OR #30 OR #31 OR #32 OR #33 OR #34 OR #35 OR #36 OR #37 OR #38 OR #39 OR #40 OR #41 OR #42	69,302
	#44	MeSH descriptor: [Fetal Macrosomia] explode all trees	135
	#45	MeSH descriptor: [Fetal Diseases] explode all trees	1,099
	#46	(Fetal Macrosomia):ti,ab,kw	347
	#47	(Macrosom*):ti,ab,kw	655
	#48	(Fetal overgrowth):ti,ab,kw	24
	#49	(large-for-gestational-age):ti,ab,kw	351
	#50	(Fetal complication*):ti,ab,kw	3,212
	#51	(Fetal disease*):ti,ab,kw	2,413
	#52	#44 OR #45 OR #46 #47 OR #48 OR #49 OR #50 OR #51	5,662
	#53	#43 AND #52	2,761
	#54	MeSH descriptor: [Americas] explode all trees 27162	27,162
	#55	MeSH descriptor: [Latin America] explode all trees	127
	#56	MeSH descriptor: [Hispanic Americans] explode all trees	1,475
	#57	MeSH descriptor: [Central America] explode all trees	288
	#58	MeSH descriptor: [South America] explode all trees	2,662
	#59	MeSH descriptor: [Caribbean Region] explode all trees	407
	#60	MeSH descriptor: [West Indies] explode all trees	389
	#61	MeSH descriptor: [Indians, South American] explode all trees	12
	#62	MeSH descriptor: [Indians, Central American] explode all trees	1
	#63	MeSH descriptor: [Mexico] explode all trees	661
	#64	(Amerindian* OR “Indians” OR “American Indian*” OR “Native America*” OR patagoni* OR “andes” OR Andean* OR amazon* OR argentin* OR Bolivia* OR Brazil* OR Brasil* OR Colombia* OR Chile* OR Ecuador* OR Ecuator* OR Guiana* OR “Guyana” OR “French Guiana” OR Guyan* OR Paraguay* OR Peru* OR Surinam* OR “Suriname” OR Uruguay* OR “Venezuela” OR Belize* OR “Costa Ric*” OR Costarric* OR Costaric* OR Salvador* OR Guatamal* OR Hondur* OR Nicaragu* OR Panam* OR Mexic* OR Mejic* OR Aruba* OR “Caribbean Netherland*” OR Curacao* OR “Sint Maarten” OR Guadeloup* OR Martiniqu* OR “Panama Canal Zone” OR “Antigua and Barbuda” OR Baham* OR Barbad* OR “British Virgin Island” OR “Cayman Island*” OR Grenad* OR “Saint Kitts and Nevis” OR “Saint Lucia*” OR “Saint Vincent and the Grenadines” OR “Trinidad and Tobago” OR “Turks and Caicos Island” OR “United States Virgin Islands” OR Anguill* OR “Leeward Island*” OR Montserrat* OR “Windward Island*” OR Cuba* OR Dominic* OR “Dominican Republic” OR Haiti* OR Jamaic* OR “Puerto Ric*” OR Puertorric* OR Puertoric):ti,ab,kw	19,858
	#65	#54 OR #55 OR #56 OR #57 OR #58 OR #59 OR #60 OR #61 OR #62 OR #63 OR #64	43,671
	#66	#53 AND #65	227
**Database**	**Scielo** **Date: November 4, 21**		**Results**
**Search Strategy**	#1	((ti:((Postpartum Hemorrhage) OR (Shock, Hemorrhagic) OR (Uterine Inertia) OR (Uterine Hemorrhage) OR (Abruptio Placentae) OR (Blood Loss, Surgical) OR (Blood Transfusion) OR (Placenta Accreta) OR (Placenta Previa) OR (uterine inversion) OR (Uterine Artery Embolization) OR (Uterine Contraction) OR (Obstetric Labor Complications) OR (Postpartum Hemorrhag*) OR (Postpartum Haemorrhag*) OR (post-partum hemorrhage) OR (post-partum hemorrhage) OR (PPH) OR (Abruptio Placentae) OR (abruption) OR (blood loss*) OR (blood transfusion) OR (placenta accreta) OR (placenta previa) OR (shock) OR (placenta praevia) OR (placental previa) OR (placental praevia) OR (atony) OR (atonic) OR (atonic uterus) OR (uterine atony) OR (uterine inertia) OR (uterine bleeding) OR (uterine hemorrhage) OR (uterine hemorrhage) OR (uterus inversion) OR (uterine artery embolization) OR (uterine contraction) OR (labor complication*) OR (labor complication*) OR (delivery complication*) OR (Hemorragia Posparto) OR (Hemorragia Pós-Parto) OR (Choque Hemorrágico) OR (Placenta Previa) OR (Placenta Prévia) OR (Contracción Uterina) OR (Contração Uterina))) OR (ab:((Postpartum Hemorrhage) OR (Shock, Hemorrhagic) OR (Uterine Inertia) OR (Uterine Hemorrhage) OR (Abruptio Placentae) OR (Blood Loss, Surgical) OR (Blood Transfusion) OR (Placenta Accreta) OR (Placenta Previa) OR (uterine inversion) OR (Uterine Artery Embolization) OR (Uterine Contraction) OR (Obstetric Labor Complications) OR (Postpartum Hemorrhag*) OR (Postpartum Haemorrhag*) OR (post partum hemorrhage) OR (post-partum hemorrhage) OR (PPH) OR (Abruptio Placentae) OR (abruption) OR (blood loss*) OR (blood transfusion) OR (placenta accreta) OR (placenta previa) OR (shock) OR (placenta praevia) OR (placental previa) OR (placental praevia) OR (atony) OR (atonic) OR (atonic uterus) OR (uterine atony) OR (uterine inertia) OR (uterine bleeding) OR (uterine hemorrhage) OR (uterine hemorrhage) OR (uterus inversion) OR (uterine artery embolization) OR (uterine contraction) OR (labor complication*) OR (labor complication*) OR (delivery complication*) OR (Hemorragia Posparto) OR (Hemorragia Pós-Parto) OR (Choque Hemorrágico) OR (Placenta Previa) OR (Placenta Prévia) OR (Contracción Uterina) OR (Contração Uterina)))) AND ((ti:((Fetal Macrosomia) OR (Fetal Disease*) OR (Fetal Macrosomia) OR (Macrosom*) OR (fetal overgrowth) OR (large-for-gestational-age) OR (Fetal complication*) OR (Macrosomía Fetal) OR (Macrossomia Fetal))) OR (ab:((Fetal Macrosomia) OR (Fetal Disease*) OR (Fetal Macrosomia) OR (Macrosom*) OR (fetal overgrowth) OR (large-for-gestational-age) OR (Fetal complication*) OR (Macrosomía Fetal) OR (Macrossomia Fetal))) OR (ab:((Fetal Macrosomia) OR (Fetal Disease*) OR (Fetal Macrosomia) OR (Macrosom*) OR (fetal overgrowth) OR (large-for-gestational-age) OR (Fetal complication*) OR (Macrosomía Fetal) OR (Macrossomia Fetal))) OR (ab:((Fetal Macrosomia) OR (Fetal Disease*) OR (Fetal Macrosomia) OR (Macrosom*) OR (fetal overgrowth) OR (large-for-gestational-age) OR (Fetal complication*) OR (Macrosomía Fetal) OR (Macrossomia Fetal))))	307
**Database**	**LILACS** **Date:** November 4, 2021		**Results**
**Search Strategy**	#1	(mh:(Postpartum Hemorrhage) OR Hemorragia Posparto OR Hemorragia Pós-Parto OR mh:(Shock, Hemorrhagic) OR Choque Hemorrágico OR Uterine Inertia OR mh:(Uterine Hemorrhage) OR Hemorragia Uterina OR mh:(Abruptio Placentae) OR Desprendimiento Prematuro de la Placenta OR Descolamento Prematuro da Placenta OR mh:(Blood Loss, Surgical) OR mh:(Blood Transfusion) OR mh:(Placenta Accreta) OR mh:(Placenta Previa) OR mh:(uterine inversion) OR mh:(Uterine Artery Embolization) OR mh:(Obstetric Labor Complications) OR Postpartum Hemorrhag$ OR Postpartum Haemorrhag$ OR post-partum hemorrhage OR post-partum hemorrhage OR PPH OR abruption OR blood loss$ OR placental previa OR placental praevia OR placenta praevia OR atony OR atonic OR atonic uterus OR uterine atony OR atoni$ OR uterine inertia OR uterine bleeding OR sangrado uterino OR sangramento uterino OR uterine hemorrhage OR uterine hemorrhage OR labor complication$ OR labor complication$ OR delivery complication$) AND (mh:(Fetal Macrosomia) OR Macrosomía Fetal OR Macrossomia Fetal OR mh:(Fetal Disease) OR Enfermedades fetales OR Doenças Fetais OR Fetal Macrosom$ OR Macrosom$ OR fetal overgrowth OR large-for-gestational-age OR Fetal complication$)	39

Inclusion criteria were: (a) case–control studies, and (b) cohorts that provided the number of PPH and FM cases. Articles were excluded if they: (a) lacked information on the number of cases with PPH and/or FM; (b) included a population of women who were not from LAC; (c) were not published in English, Spanish, or Portuguese; and (d) included articles with a different design (i.e., editorials, review articles).

All studies identified in the search and that met the inclusion criteria underwent an independent assessment by two review authors of the titles and abstracts using the Rayyan web application. 13 Discrepancies during the evaluation were resolved by a third author. All papers that passed the first phase were fully read and evaluated by two authors independently. Disagreements between the two authors on the selection of studies were resolved by a third author.

The outcome variable of interest was PPH, which was defined as blood loss greater than or equal to 500 ml, whereas severe PPH was considered as blood loss greater than or equal to 1,000 ml or when the blood loss caused hemodynamic instability and/or signs or symptoms of hypovolemia. 3 Furthermore, FM was defined as a fetal birth weight greater than or equal to 4,000 g or greater than the 90th percentile for the gestational age reported in each study. 7

Data extraction was performed independently by two authors using Excel (Microsoft Corp., Redmond, WA, USA), and data accuracy was evaluated by a third author. For the extraction, a pilot test of 5 articles was performed. After the inclusion of additional items, the authors collected the following information: first author, year of publication, period of data collection, country, journal, title, setting, objective, selection criteria, age of the women, sample size, operational definition of PPH and FM, number of PPH and FM cases, estimated risk ratio (RR) or odds ratio (OR) with its respective confidence interval (CI), statistical test used, and conclusions.

The Newcastle-Ottawa scale (NOS) was used to assess the quality of the studies. 14 This assessment was performed independently by two authors with a final consensus by a third author.

The characteristics of the studies included were described using data extraction performed in Excel (Microsoft Corp.). For the studies included, the *meta esize* command of the Stata 17 statistical program (StataCorp LLC., College Station, TX, USA) was used to calculate the effect sizes of the binary summary data (OR). Then the overall effect size was estimated along with the 95% CI using the *meta summarize* command. 15 When a study did not report the OR, it was calculated using the *csi* command. To evaluate the heterogeneity of the studies, the I ^2^ statistic was used, with values of 25, 50, and 75% being considered as low, moderate, and high heterogeneity, respectively. 16 The studies' findings were illustrated in the form of a forest plot. Publication bias was not assessed because the meta-analyses were performed with fewer than ten studies, as recommended in the Cochrane handbook. 17

The leave-one-out method was used as a sensitivity analysis, excluding one study at a time to verify the stability of the results and the sources of heterogeneity.

Ethics committee approval was not sought because the data from the studies are public domain, which precludes identification of the participants in each study.

## Results

**Fig. 1:**
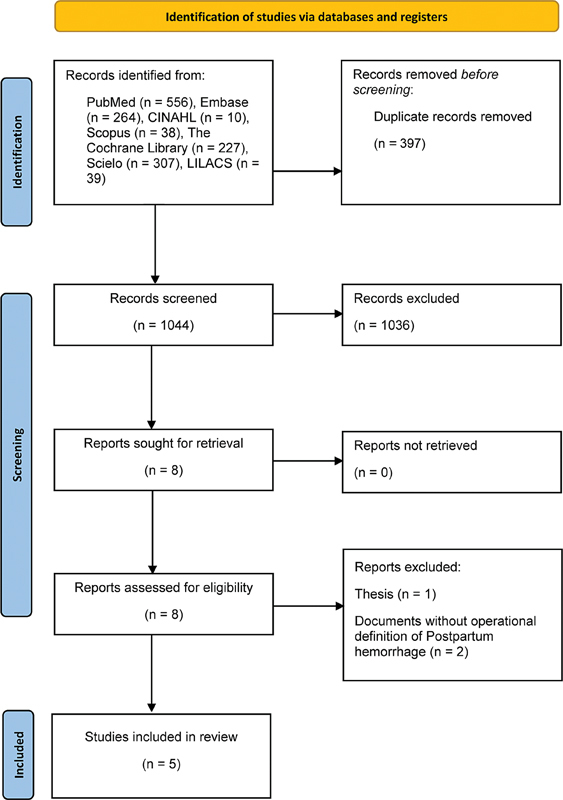
PRISMA 2020 flow diagram of study selection.

A total of 1,044 articles were evaluated by title and abstract, 8 of which were eligible for full-text evaluation. Of these articles, 5 met the selection criteria and were included in the present systematic review ( Fig. 1 ). The 3 excluded articles were due to being a thesis published in a repository and, thus, it had not been evaluated in a peer review process, which is a quality standard recognized by the scientific community, 18 while the other two 19 20 were excluded due to not having operationally defined the PPH variable ( Chart 2 ).

**Chart 2:** List of excluded studies

**Study**		**Reason**
1	Machado, O., 2017. [Factors associated with uterine atonia in the postwork of the Hospital Uldarico Rocca Fernández in Villa el Salvador, in the period January - December 2014]. Repository of the San Martín de Porres University. https://repositorio.usmp.edu.pe/handle/20.500.12727/2684?locale-attribute=de	The study is a thesis published in a repository.
2	Salazar de Dugarte G, González de Chirivella X, Faneite Antique P. Incidencia y factores de riesgo de macrosomía fetal. Rev Obstet Ginecol Venezuela. 2004; 64(1):15–21.	Does not operationally define the variable postpartum hemorrhage.
3	Galarza MP de L. Indicadores clínico epidemiológicos y materno-fetales de atonía uterina en puérperas post cesárea primaria en una clínica privada de agosto 2017–agosto 2018. Rev Fac Med Humana. 2019; 19(2):7–7.	Does not operationally define the variable postpartum hemorrhage.

The studies included were published between 2003 and 2020. One was considered multi-country because it was conducted in Argentina and Uruguay, 21 one was performed in West Indies, 22 one in Antigua and Barbuda, 23 one in the French Guyana, 24 and one in Suriname. 25 Regarding the design of the studies, one was a cohort study, 21 and the rest had a case-control design. Regarding the context of participant recruitment, three studies were conducted in a single hospital, 22 23 24 and two were conducted in more than one setting. 21 25 Regarding the characteristics of the population included in the selected studies, the reporting of participants' age was heterogeneous; the age range included does not allow specification of the upper limit but does allow specification of the lower limit, so none of the studies included patients younger than 12-years-old. Also, due to the data collection process, all studies were based on medical record review. The studies' characteristics are summarized in Chart 3 .

**Chart 3:** Characteristics of the studies included

**Author (year)**	**Country(ies)**	**Study design**	**Data collection period**	**Setting**	**Age (years), in mean (SD) or range**	**Number of women with PPH**	**Number of women with severe PPH**	**Sample size (n)**	**Quality assessment (NOS)**
										Sosa et al., 2009	Argentina, Uruguay	Cohort	Data collected during 3 periods, but only 2 were used. First period: October to December 2003 Third period: October to December 2005	Hospital	NR	1,221	309	11,323	8
Richardson et al., 2017	West Indies	Case-control	January 2007 to December 2009	Hospital	29.84 (6.1)	50	NE	316 cases; 316 controls	6
Martin et al., 2003	Antigua and Barbuda	Case-control	July 1991 to January 1997	Hospital	27.9	52	NE	157 cases; 157 controls	6
Firmin et al., 2019	French Guiana	Case-control	September 2014 to September 2015	Maternity Department of Hospital	NR	154	39	154 cases; 308 controls	8
Kodan et al., 2020	Suriname	Case-control	January to December 2017	Hospital	12–35 and > 35	585	216	8747	6

**Abbreviations:** PPH, postpartum hemorrhage; NOS, Newcastle-Otawa scale; NR, not reported; NE, not evaluated; SD, standard deviation.

**Fig. 2:**
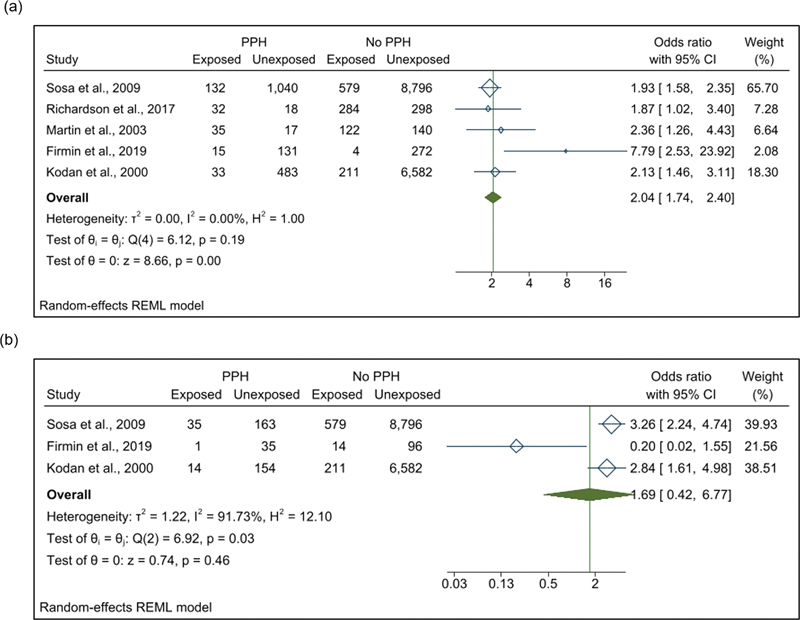
Forest plot showing the pooled odds ratio of association between postpartum hemorrhage (a), severe postpartum hemorrhage (b) and fetal macrosomia. **Abbreviations:** CI, confidence interval; PPH, postpartum hemorrhage.

**Fig. 3:**
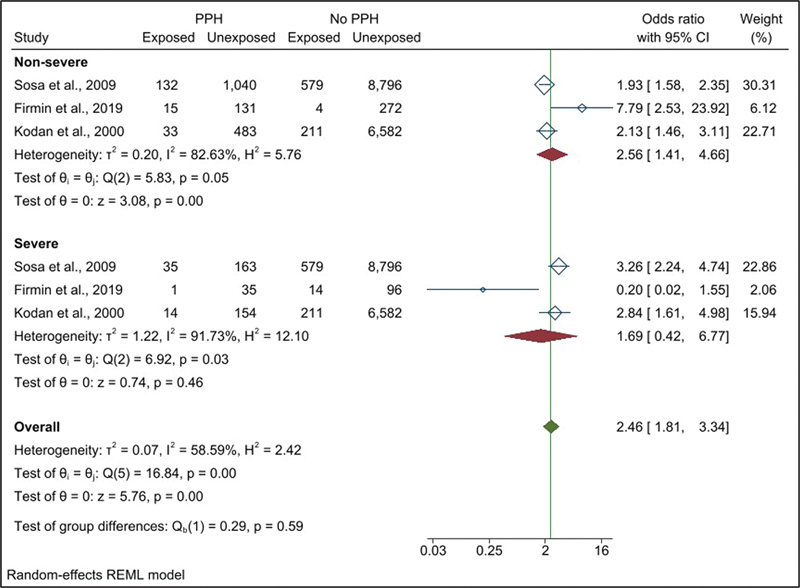
Forest plots showing the pooled odds ratio for fetal macrosomia according to the severity of postpartum hemorrhage in studies measuring severe postpartum hemorrhage. **Abbreviations:** CI, confidence interval; PPH, postpartum hemorrhage.

Regarding the operational definition of PPH, most studies presented a similar definition for this entity. All the included studies define it as a blood loss greater than or equal to 500 ml after delivery. Of the five studies, only three included the operational definition of severe PPH. Sosa et al., 21 defined it as blood loss greater than or equal to 1,000 ml; Firmin et al., 24 as the loss of at least 4 g/dl of hemoglobin, or the need for transfusion of at least four packs of red cell concentrates (RCC), the need for surgery, and/or maternal death; and Kodan et al., 25 as blood loss of at least 1,000 ml, bleeding associated with arterial hypotension, or the need to transfuse at least three RCC packs. Concerning the operational definition of FM, all studies define it as a birth weight greater than or equal to 4,000g. The pooled OR of FM cases reporting PPH in the LAC region, calculated from a meta-analysis of 5 eligible studies, was 2.10 (95% CI: 1.79–2.47), with low heterogeneity described between studies (I ^2^ : 0%) ( Fig. 2a ). The meta-analysis of severe PPH (≥1,000 ml of postpartum blood loss) was performed with three studies by measuring the presence of this outcome. 21 24 25 The pooled OR of severe PPH was 1.61 (95% CI: 0.40–6.48), with high heterogeneity among studies (I ^2^ : 91.89%) ( Fig. 2b ). A subgroup analysis according to the severity of PPH only for the studies that measured severe PPH is presented in Fig. 3 , with the pooled OR for nonsevere cases being 2.68 (95% CI: 1.43–5.04), pooled OR of 1.61 (95% CI: 0.40–6.48) for the severe PPH, and a total pooled OR of 2.46 (95% CI: 1.84–3.27) ( Fig. 3 ).

**Fig. 4:**
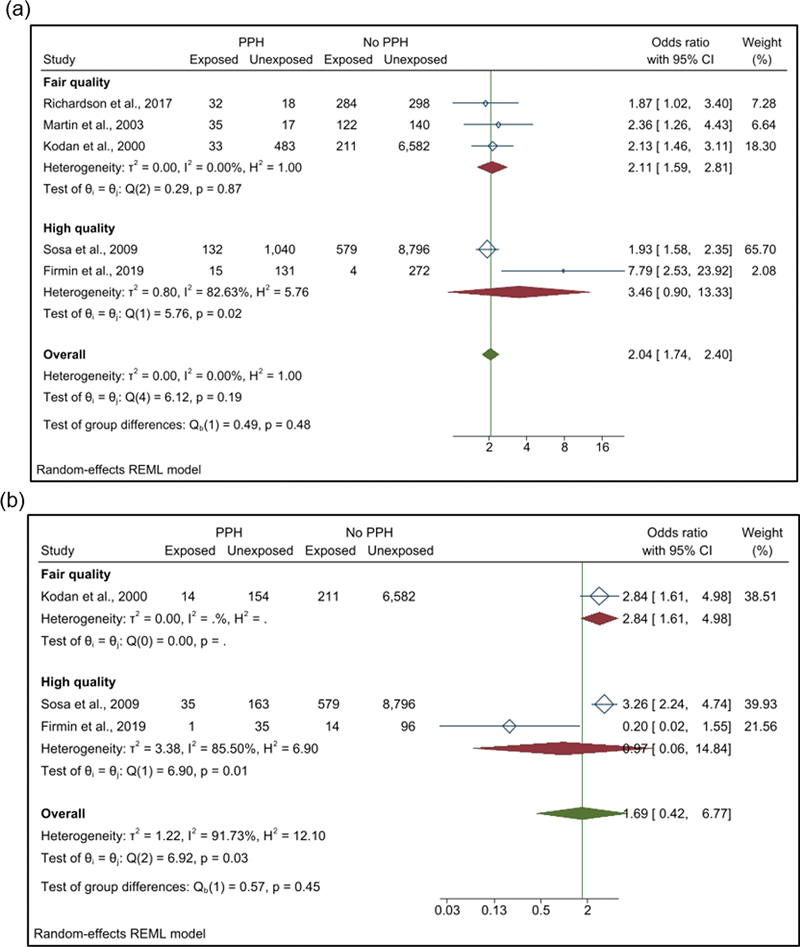
Subgroup analysis according to the quality of the included studies (a) association between postpartum hemorrhage and fetal macrosomia; (b) association between severe postpartum hemorrhage and fetal macrosomia. **Abbreviations:** CI, confidence interval; PPH, postpartum hemorrhage.

In relation to the quality of the studies included, according to the modified NOS, two 21 24 were of high quality (score of 7–9 points) and three were of fair quality 22 23 25 (score of 4–6 points) ( Chart 4 ). Additionally, we estimated the association between PPH and FM, and severe PPH and FM. In relation to the studies with fair quality, a pooled OR of 2.14 (95% CI: 1.61–2.84; I ^2^ : 0%) was observed for PPH, while the high-quality studies had a pooled OR of 3.62 (95% CI: 0.92–14.21; I ^2^ : 83.13%) and 0.96 (95% CI: 0.06–15.36; I ^2^ : 86.00%) for common and severe PPH, respectively, presenting high heterogeneity in both cases ( Fig. 4 a e b ).

**Chart 4:** Quality assessment of the included studies

N°	Year	Author	Journal	Title	Criteria								Total
					Selection				Comparability	Outcome			
					1	2	3	4	1	1	2	3	
1	2009	Sosa et al.	Obstetrics & Gynecology	Risk Factors for Postpartum Hemorrhage in Vaginal Deliveries in a Latin-American Population	*	*	*	–	**	*	*	*	8
2	2017	Richardson et al.	West Indian Medical Journal	Outcome of macrosomic infants at the university hospital of the West Indies	*	*	*	*	–	*	*		6
3	2003	Martin et al.	West Indian Medical Journal	A Case Control Study of the Prevalence of Perinatal Complications Associated with Fetal Macrosomia in Antigua and Barbuda	*	*	*	*	–	*	*		6
4	2019	Firmin et al.	Journal of Gynecology Obstetrics and Human Reproduction	Postpartum hemorrhage: incidence, risk factors, and causes in Western French Guiana	*	*	*	*	**	*	*		8
5	2020	Kodan et al.	PLoS ONE	Postpartum hemorrhage in Suriname: A national descriptive study of hospital births and an audit of case management	*	*	–	–	**	*	*		6

**Abbreviations:** NOS, Newcastle-Otawa scale. **Note:** The modified Newcastle-Ottawa scale (NOS) was used to assess the quality of the studies included.

**Fig. 5:**
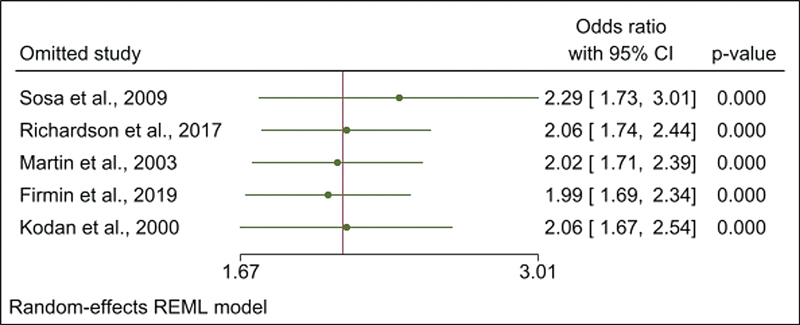
Leave-one-out sensitivity analysis of the pooled OR of association between postpartum hemorrhage and fetal macrosomia. **Abbreviations:** CI, confidence interval.

**Fig. 6:**
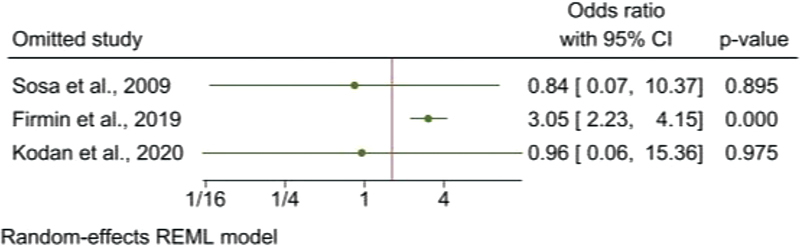
Leave-one-out sensitivity analysis of the pooled OR of association between severe postpartum hemorrhage and fetal macrosomia. **Abbreviations:** CI, confidence interval.

The sensitivity analysis consisted of the leave-one-out method showing pooled OR values of 2.32 (95% CI: 1.76–3.06) with the exclusion of the study by Sosa et al. 21 and 2.12 (95% CI: 1.79–2.51) excluding the study of Richardson et al. 22 These values were within the estimated CI of the combined OR with all the studies to estimate the association between PPH and FM ( Fig. 5 ). For severe PPH, the sensitivity analysis showed combined OR values of 3.05 (95% CI: 2.23–4.15) with the exclusion of the study by Firmin et al., 24 and 0.96 (95% CI: 0.06–15.36) with the exclusion of the study by Kodan et al., 25 being a value that was not within the estimated CI of the combined OR with all the studies ( Fig. 6 ).

## Discussion

The present study sought to determine the association between FM and PPH in LAC women. A total of 5 articles met the eligibility criteria established for this systematic review. The meta-analysis performed showed that FM is a risk factor for PPH in pregnant women in LAC, with low heterogeneity among the studies in this analysis. Regarding the associations between FM and severe PPH, only 3 studies were found, and the meta-analysis showed no association between these clinical conditions, although there was high heterogeneity.

The pooled analysis showed that FM cases in LAC were more likely to develop PPH compared with deliveries in which FM was not present. This result was similar to a previous systematic review conducted in Asian, European, and African populations (OR: 2.05; 95% CI: 1.90–2.22). Thus, the strength and direction of the association between FM and PPH found in LAC are consistent with those of other regions of the world. Regarding the mechanism that would explain this relationship, it is postulated that uterine overdistension is the main mechanism of the relationship between the two variables. 26 It is also described that a larger placental size could increase the surface area for postpartum bleeding and, thus, the risk of PPH. 27 In LAC, there has been an increase in cases of FM in recent decades, possibly explained by the increase in the prevalence of obesity among women. 9 The increase in the prevalence of FM in LAC requires timely diagnosis and appropriate medical management, as well as protocols for the care of women who may present complications, such as PPH, related to the presence of FM. Also, during the third stage of labor, the utility of using oxytocin or other uterotonics to prevent PPH (prophylactic use) is described. 28 Only three included studies reported data about using oxytocin or other uterotonics to prevent it. 21 24 25 However, these data are not sufficiently presented to obtain the proportion of use of prophylactic uterotonics according to the presence of PPH or FM. As such, while it was not possible to describe or evaluate the influence of prophylaxis measures in these cases, it is reasonable to think these measures may influence bleeding. Hence, future studies could evaluate the influence of prophylactic uterotonics for PPH in FM cases.

The studies included in this review were conducted in South and Central American countries. Although no data are available regarding the association of PPH and FM in other LAC countries, similar results are expected to be found among the different countries of this region since they are mostly low- and middle-income countries. Globally, LAC is one of the regions with the highest maternal mortality rates, and in this regard, PPH is an important public health problem. Although there was a reduction in deaths in both Latin America (124 to 69 per 100,000 live births) and the Caribbean (276 to 175 per 100,000 live births) from 1990 to 2015, the expected target of a 75% reduction in maternal mortality described in the Millennium Development Goals was not achieved. 29 While the main causes of maternal death are preventable, including PPH, inequalities in access to health services and quality of care received by women in this region of the world explain why PPH continues to be the leading cause of maternal death in LAC countries. 8 There is a need for proper identification of cases of FM during clinical management, and to ensure that the necessary resources are available for possible complicated deliveries, including cases of PPH. The improvement and implementation of programs to detect and prevent early factors that may condition the presence of PPH and other maternal complications are also needed. 30

Regarding severe PPH, only three studies could be included to evaluate this outcome and its association with FM, because the remaining studies did not report the number of cases of PPH greater than or equal to 1,000 ml. No association was found between FM and the presence of severe PPH. The low number of studies that evaluated this outcome, as well as the high heterogeneity among these studies, could explain the lack of association between FM and severe PPH in the meta-analysis and the sensitivity analysis, reaffirming what was described. Since FM was found to be associated with PPH, it would be expected that severe PPH would also be related to this clinical condition. A few studies in the literature have evaluated and described an association between FM and severe PPH. 4 31 Thus, future studies of adequate methodological rigor are needed to evaluate the association between FM and severe PPH in LAC. Defining this association would be useful to emphasize the need for timely identification of FM cases during the care of pregnant women to reduce the risk of maternal complications and mortality in this region.

Some limitations should be considered in the interpretation of the results of this systematic review. The first limitation is due to the high number of covariates used in the included studies as confounding variables, which makes it impossible to perform a meta-regression to evaluate the association between PPH and FM adjusting for variables that are relevant in the point estimate. The second limitation is the presence of high methodological heterogeneity found in the second analysis (association of FM and severe PPH), which does not allow acceptable certainty regarding the estimate obtained for this association, although the literature reports an association between these two clinical conditions. This lack of certainty prevents a conclusive analysis, requiring new evaluations including studies with less heterogeneity. The third limitation lies in the scarcity of information, since only studies in populations from less than half of the LAC countries were found. Although it is expected to find similar results in these other countries, the generalization of these results should be considered with caution.

Despite the aforementioned limitations, the present study's preparation rigorously followed the updated PRISMA 2020 guidelines for systematic reviews. 11 Furthermore, sensitivity analysis and subgroup analysis were conducted to strengthen the conclusions and credibility of the findings. Additionally, each article included was evaluated according to the criteria of the NOS. Therefore, we consider the assessment of the association of interest in the LAC population to be adequate.

## Conclusion

In conclusion, the results of this systematic review indicate that FM is related to PPH in the LAC population. The evidence available to date included the evaluation of this association in only some LAC countries, with results in line with the current scientific literature.

Regarding FM and its association with severe PPH, further research following a rigorous study design and measurement of severe PPH are required to evaluate this association. Adequate identification of FM as well as the implementation or improvement of maternal health services, including more human resources prepared for the care of obstetric emergencies, as well as appropriate resources and infrastructure for the care or transfer of patients presenting PPH are necessary for the management of this health problem in LAC. Likewise, raising awareness and training health personnel to identify patients with PPH is of vital importance to prevent complications associated with this condition, as well as for better decision making and improved quality of care.
